# Intermittent preventive treatment of malaria during pregnancy: a qualitative study of knowledge, attitudes and practices of district health managers, antenatal care staff and pregnant women in Korogwe District, North-Eastern Tanzania

**DOI:** 10.1186/1475-2875-4-31

**Published:** 2005-07-20

**Authors:** Godfrey Mubyazi, Paul Bloch, Mathias Kamugisha, Andrew Kitua, Jasper Ijumba

**Affiliations:** 1 National Institute for Medical Research, Ubwari Research Station, P.O. Box 81, Muheza, Tanzania; 2 DBL-Institute for Health Research and Development, Jaegersborg Allé 1 D, DK-2920, Charlottenlund, Denmark; 3 Amani Medical Research Centre, P.O. Box 4, Amani, Tanzania; 4 National Institute for Medical Research, Headquarters, P.O. Box 9653, Dar es Salaam, Tanzania; 5 Centre for Enhancement of Effective Malaria Interventions, P.O. Box 9653, Dar es Salaam, Tanzania; 6 University of Dar es Salaam, Department of Zoology, P.O. Box 35064, Dar es Salaam, Tanzania

## Abstract

**Background:**

Intermittent preventive treatment of malaria during pregnancy (IPTp) is a key intervention in the national strategy for malaria control in Tanzania. SP, the current drug of choice, is recommended to be administered in the second and third trimesters of pregnancy during antenatal care (ANC) visits. To allow for a proper design of planned scaling up of IPT services in Tanzania it is useful to understand the IPTp strategy's acceptability to health managers, ANC service providers and pregnant women. This study assesses the knowledge, attitudes and practices of these groups in relation to malaria control with emphasis on IPTp services.

**Methods:**

The study was conducted in February 2004, in Korogwe District, Tanzania. It involved in-depth interviews with the district medical officer (DMO), district hospital medical officer in charge and relevant health service staff at two peripheral dispensaries, and separate focus group discussions (FGDs) with district Council Health Management Team members at district level and pregnant women at dispensary and community levels.

**Results:**

Knowledge of malaria risks during pregnancy was high among pregnant women although some women did not associate coma and convulsions with malaria. Contacting traditional healers and self-medication with local herbs for malaria management was reported to be common. Pregnant women and ANC staff were generally aware of SP as the drug recommended for IPTp, albeit some nurses and the majority of pregnant women expressed concern about the use of SP during pregnancy. Some pregnant women testified that sometimes ANC staff allow the women to swallow SP tablets at home which gives a room for some women to throw away SP tablets after leaving the clinic. The DMO was sceptical about health workers' compliance with the direct observed therapy in administering SP for IPTp due to a shortage of clean water and cups at ANC clinics. Intensified sensitization of pregnant women about the benefits of IPTp was suggested by the study participants as an important approach for improving IPTp compliance.

**Conclusion:**

The successful implementation of the IPTp strategy in Tanzania depends on the proper planning of, and support to, the training of health staff and sustained sensitization of pregnant women at health facility and community levels about the benefits of IPTp for the women and their unborn babies.

## Background

Malaria continues to be a major public health burden in Tanzania, a country with the world's third largest population at risk of stable malaria, after Nigeria and the Democratic Republic of Congo [1]. About 35 million Tanzania's population are at risk, pregnant women and under five children being the most vulnerable groups [1,2]. The NMCP's Mid-term Strategic Plan for 2002–2007 reports that malaria account for about 1.3% reduction in national economic growth, 30% of the national disease burden. There are about 1.7 million cases per year among of the pregnant women alone. One recent study in northern Tanzania reported malaria to be responsible for about 20% of all deaths among pregnant women [3], while malaria related anaemia contributes significantly to maternal deaths in Tanzania [4].

For decades chloroquine (CQ) was the first line drug for the treatment of uncomplicated malaria in Tanzania [5,6]. Accumulating evidence of increasing parasite resistance to CQ and treatment failure rates prompted the Ministry of Health (MOH) to replace CQ with SP as the first line in 2001 [1,7-10]. SP has also been recommended for IPTp purposes. According to national policy guidelines its administration at ANC clinics was supposed to start since then, however, appropriate training of service givers and community sensitization were to be effectively done thereafter in most districts (Dr. Pasiens Mapunda, CEEMI Director and Dr. M.W. Marero-NMCP, personal communication). In the light of increasing parasite resistance, the policy recommendation of SP for preventive and curative treatment against malaria was left as an interim strategy while an appropriate combination therapy is being considered [7].

In an attempt to achieve the targets of the Abuja Declaration of 2000 regarding the reduction of malaria burden, many sub-Sahara African countries (including Tanzania) have targeted to reduce the burden of malaria through IPTp and insecticide-treated nets [2,11]. Under the IPTp strategy it is recommended that all pregnant women in malaria endemic areas receive a full three tablets single dose of SP at least twice during the second and third trimester of pregnancy. ANC clinics are considered an important entry points to target the pregnant women as records show that about 80% of Tanzanian pregnant women attend such clinic at least once during their pregnancy [12,28], although the latter figure contradicts the 98% attendance rate reported from another source [40]. Nevertheless, the extent to which public and private facilities will comply with the IPTp guidelines remains to be seen [12].

While empirical evidence from Kenya [13,14] and Malawi [15,16,18,19] indicate high efficacy of IPTp in reducing anaemia during pregnancy and increasing birth weight, reports on treatment failures and parasite resistance to SP in malaria endemic countries has stimulated debates about the appropriateness of SP [10,20,22] and has prompted some African NMCPs to recommend combination therapy [21,23]. Depending on further scientific documentation on safety and efficacy in pregnancy, artemisinin-based combination therapy (ACT) may be a useful alternative to SP in the future [12]. However, there are critical debates about the potential limitations of ACT in terms of cost to the poor, its complicated treatment regimen and the limited knowledge about acceptability among users [20,24-26,28]. The UNICEF-UNDP-World Bank-WHO Special Programme for Research and Training in Tropical Diseases (TDR) has warned that an intervention deemed efficacious within clinical trials may not be easily transformed into the reality of control operations [22].

To reduce maternal morbidity and morality and better health for the baby, focused ANC package advocates for the timely and appropriate care during pregnancy and timely attendance at ANC clinics is a key factor for the effective delivery of IPTp services [30]. However, inadequate/irregular attendance has been noted in some sub-Sahara African countries [29]. In Tanzania, only 40% of pregnant women deliver at health facilities although some records show a high antenatal clinic attendance rate [40]. One study in Kenya found the late timing of the first dose of SP corresponding with late registration at ANC clinics among pregnant women [17]. Another study from the same country found that despite high awareness about the IPTp strategy, only 5% of pregnant women had received two or more doses of SP as preventive treatment and only 14% of the women received at least one dose [18]. Similarly findings were reported from Malawi whereby less than 40% of the 391 pregnant women surveyed in Blantyre district received the full dose regimen of SP for IPTp [17]. In two cross-sectional studies funded by the World Health Organization in Muheza district, Tanzania (Massaga *et al*, personal communication) and Mpwapwa district in Central Tanzania (Magesa *et al*, personal comunication), low compliance with the use of SP was partly attributed to health care providers' and users' fear of side effects of SP and their inadequate knowledge of the correct dose. NMCP's Mid-Term Strategic Plan for 2002–2007 states that serious side effects of SP are rare and that fear among the users has been aggravated by speculative media reports.

The present study was carried out in Korogwe District, Tanzania in February 2004 and assessed (a) the extent to which staff (clinical officers and nurses) at public health facilities and pregnant women understood and appreciated the IPTp strategy, (b) district-level health managers' opinion of the appropriateness of the IPTp strategy and potential impediments for its effective implementation at the district level and (c) the opinion of each of these study populations regarding programmatic changes that could improve the delivery of ANC services and increase compliance with IPTp at ANC clinics.

## Methods

### Study Design

This pilot study was cross-sectional in nature and was part of the activities scheduled for the rapid assessment of acceptability and viability of the IPTp strategy at district level using data collection tools developed by The Centre for Enhancement of Effective Malaria Interventions (CEEMI), Dar es Salaam. The tools would be refined where necessary to be adopted in the main study scheduled to be conducted later in Muheza district. The latter district has a record of *P. falciparum *resistance to SP [7,9,39]. Korogwe was selected for this pilot study because of her being close to Muheza and likeliness to have similar drug resistance and socio-economic characteristics to those in Muheza. Since it was a pilot survey and given the limited study time frame, it was seen feasible to cover two rural government dispensaries with the view that the main study would cover more health facilities – government and non-government alike from both rural and urban/peri-urban settings.

### Study area

The study was carried out in Korogwe District in Tanga Region, North-Eastern Tanzania. The district has four divisions, namely, Bungu, Korogwe, Magoma and Mombo, 20 wards and 133 villages and an area of 3,756 km^2^. The district shares borders with Muheza, Lushoto and Handeni districts to the East, North and south-west, respectively. There are three hospitals (one owned by the government and two by the Church), four rural government health centres, 47 dispensaries and 16 village health posts. The National Census (2002) shows the district to have 261,004 population and 1.4% annual population growth rate. The main tribes are the *Zigua*, *Sambaa *and *Bondei*. Small-scale farming of food crops, mainly maize, rice, bananas and beans and cash crops such as coffee, cashew nuts, tea, oranges, cardamon are the main economic activity of the majority of the residents.

### Targeted health facilities and villages

Two government dispensaries, Ngombezi and Kwamsisi, were selected for the study and are located far from each other. Within the catchment area of the two dispensaries, three wards were selected, based mainly on accessibility. Finally, four villages were randomly selected from the following wards: Mgombezi Ward (Kitifu and Tabora villages), Korogwe Ward (Kwakombo village) and Kwamsisi Ward (Kwamsisi village).

### Study populations

The study involved district-level health officers who were members of the District Council Health Management Team (CHMT), dispensary-based health staff responsible for ANC services and pregnant women eligible for ANC services. A total of 11 district CHMT members and managers participated as key informants in the study. These were the DMO, District Hospital Medical Officer In-charge (DHMOI), District Nursing Officer, District Health Secretary, District Reproductive and Child Health Coordinator, District Laboratory Technologist, District Pharmacist, District Dental Officer, District School Health Coordinator, District Health Officer and a district private health facility representative.

From each of the two study dispensaries a clinical officer in-charge and two nursing staff involved in the provision of ANC services were selected to participate in the study. Six groups of pregnant women, with 8–12 participants per group, participated in focus group discussions (FGDs).

### Data collection methods

Information was collected through in-depth interviews with the DMO, the DHMOI and health facility-based staff and through separate FGDs with CHMT members and pregnant women. Separate interviews with the DMO and the DHMOI were arranged to give these senior district level medical officers an opportunity to express their experiences and opinions independently as key decision-makers by virtue of their positions. The interviews with the DMO and DHMOI, as well as FGDs with pregnant women addressed opinions about the rationale and practicability of the IPTp strategy, the availability and usefulness of the national IPTp guidelines.

Health facility-based staff were asked for their views about the strengths and weaknesses of ANC services (including those related to IPTp) and the utilization of such services by pregnant women. Assessment was also made of the knowledge, attitudes and practices of the staff in relation to malaria prevention and case management and, more specifically, the IPTp approach.

CHMT members were asked for their opinions about the acceptability of SP for IPTp and the actual or potential practical difficulties in the implementation of the IPTp strategy in the district.

Six FGDs were held for pregnant women. Two were held in Kitifu village, two in Kwakombo village, one in Tabora village and one in Kwamisi village. The FGD held in Kwamsisi village was organized at the local dispensary following ANC attendance as it was seen by local leaders as the more convenient means for mobilizing the women for the study based on the scattered nature of hamlets. FGDs in the other study villages were held at village government offices and the village government leaders helped to mobilize the FGD participants. The pregnant women were asked to give their views about the use of SP during pregnancy by pregnant women attending or eligible for antenatal care.

All the study population groups were asked for their suggestions regarding what could be done to improve ANC and IPTp services, that is, potential measures for strengthening the implementation of the IPTp strategy and enhancing its acceptability to the target users.

### Data analysis

The FGDs notes were taken by hand and then validated during transcription by listening to tape recordings made during the discussion sessions. Summary transcriptions of the handwritten notes from both FGDs and in-depth interviews were prepared every day immediately after data collection and were elaborated later in triangulation after completing the entire survey.

### Ethical considerations

The study was undertaken under approval by the National Institute for Medical Research of Tanzania (NIMR). Oral informed consent at district, ward and village levels was solicited in advance of data collection. Study populations were invited to participate and asked for their consent following an explanation about the purpose of the study. They were also told that any rejection would have no penalty to them whatsoever.

## Results

### Knowledge of and attitudes about malaria, SP and IPTp among health staff

Health personnel at both dispensary and district level were generally aware that SP is the recommended drug for preventive treatment of malaria in pregnant women. An assertive statement from a clinical officer attesting this was: "*Yes, we know through presumptive prescription of SP, that the MOH is determined to protect pregnant women, who are the most vulnerable groups together with under-fives and that they should receive it free of charge*". In-charges of the two dispensaries acknowledged having received IPTp guidelines from the MOH.

District CHMT members appreciated the 'plentiful' supply of SP at government health facilities (which was confirmed by other health service staff at dispensary level). Unlike common shortages of CQ in the past, SP is readily available at all times. The district CHMT members and dispensary staff reported occasional shortages of antipyretics (mainly paracetamol) at primary level health facilities due to over-prescription.

Most interviewees at health facility level reported having received complaints from members of the community in their respective catchment areas about SP. One clinical officer explicitly said that: "S*ome women who come to the health facility reluctantly take SP for fear of its side effects. This is an indication that there may be a significant proportion of non-compliant pregnant women who take SP tablets, but without swallowing them upon leaving the clinic*". Although the majority of clinical officers reported to have referred a few cases due to treatment failure or serious side effects, it was apparent from the discussions that patients commonly referred themselves when experiencing even mild side effects or discomforts. At the Korogwe District government hospital it was reported that only two cases had previously been admitted with SP-related adverse reactions (i.e. Steven-Johnson Syndrome). At Ngombezi Dispensary there was only one record of a patient who had developed such adverse reaction. In the course of discussion with the officer in-charge of the dispensary he asserted that: "*Only a few patients are of the opinion that if they take SP and develop adverse reactions, it is due to some additional conditions or abnormalities in their bodies i.e. besides the malaria infection*". Furthermore, it was pointed out that some patients disliked Fansidar^® ^but were prepared to take Metakelfin^®^, believing the two drugs to be different. The general feeling is that SP is synonymous with Fansidar^® ^but not Metakelfin^® ^while in reality the two drug regimens are the same.

The DHMOI argued that one reason for poor compliance with IPTp services could relate to inappropriate prescriptions. Pregnant women consulting private health facilities may opt for drugs other than SP and due to their profit motive such facilities may accept clients' demand for particular drugs even if sub-optimal. The DMO believed that negative community perceptions of SP are likely to continue reducing compliance unless there is an enabling environment for implementing the DOT strategy at ANC clinic level. Based on his experience as DMO for Korogwe District and (previously) Tunduru District (located in southern Tanzania) he believed that the DOT strategy would be effective if the problem with the shortage of clean water and cups at peripheral health facilities was solved. Furthermore, he argued that even if pregnant women were persuaded to bring along water and cups to ANC clinics, the water quality and hygienic standard would probably not improve substantially. Requesting that women bring along water and cups would also introduce inequity, as the poorest households could not afford to buy clean water and cups. In expressing these opinions, he was referring to the National ANC Training Manual for clinical health workers which stated that pregnant women should take SP under supervision of the service provider at the ANC clinics and that water, cups and facilities for washing the cups with soapy water and rinsing with clean water between use should be available at the clinics.

The CHMT members indicated that some dispensaries are located very far from the main roads and are difficult to reach even by vehicles. Besides affecting the routine health supervision activities, the long distances and transport problems are among the factors discouraging the utilization of ANC services at peripheral health facilities. These factors may induce pregnant women to consult traditional healers or traditional birth attendants, regardless of any possible risks to the mother and unborn baby that may be attributed to poor services obtained from such alternative providers.

### Malaria-related knowledge among pregnant women

Malaria during pregnancy was reported by women in Kwamsisi and Kitifu villages to contribute to: stillbirths, death before or during child delivery, delivering a child with malaria and too much bleeding during and after delivery. However, malaria was commonly known among all FGD participants as a leading communicable disease. Anaemia in under-fives and pregnant women, fever, joint pains, fatigue, loss of appetite and general body malaise were reported by the majority of the participants to be prominent malaria-related conditions. Other conditions which they associated with malaria in pregnancy were swelling of legs, dizziness, high blood pressure/high heartbeats, abortion, dehydration, headache and vomiting. In Kwamsisi village persistent menstruation, even during pregnancy, was associated with malaria. The local terms for malaria were mixed, this partly being influenced by the ethnic language of the speakers. Some respondents interchangeably used the name *homa *to mean fever and this was exclusively attributed to malaria, whereas other respondents, particularly among people of the *Pare *and *Gweno *ethnic communities, used the term *itheng'u *to mean malaria.

### Treatment seeking behaviour among pregnant women

Although the majority of women reported contacting formal health facilities for malaria diagnostic and treatment services, some (especially in Kitifu and Kwamsisi) did not feel shy about testifying that traditional health practitioners were commonly consulted for conditions such as coma and convulsions (locally popular as *degedege *or *mchango*). Self-medication with modern pharmaceuticals from retail sources (shops/kiosks) was also reported to be common.

Poor quality of healthcare services was reported to contribute to poor attendance of pregnant women at health facilities providing ANC services. Specific physical and service-related factors mentioned to be inappropriate or inadequate are summarised in Table 1. These all have potential negative implications on ANC attendance. Participants of the FGD held in Kwamsisi village expressed dissatisfaction regarding the shortage of public health staff houses, which had necessitated the use of a building intended for ANC services as a residential house for health staff. The shortage of rooms for temporarily admitting pregnant women due for delivery seemed to be a strong concern among the majority of the discussion participants and as one indicated: "*When a pregnant woman comes to the clinic and finds that another woman is being attended to, she is told to go back and come the following day or later because there is no space or delivery bed to cater for an extra patient*". She also stated that: "*We are sometimes rebuked by nurses as if we are young children*". Other women expressed dissatisfaction with the way the current ANC service staff provide information about malaria to their clients. Probed further on this issue an FGD participant at Kwamsisi asserted: "*They address you as if you have come to beg for some money from them or as if they are forced to attend you at the clinic*". Finally, staff shortages affecting the quality of services at ANC clinics and the long distances to ANC clinics affecting the accessibility of services were mentioned in the FGDs as major problems limiting the attendance of pregnant women to ANC clinics.

**Table 1:** Deficiencies and problems related to ANC services as presented and broadly supported by pregnant women during FGDs in the study villages.

**Kwamsisi village**	**Kwakombo village**	**Kitifu village**	**Tabora village**
'No laboratory facilities at the dispensary Shortage of delivery ward at the dispensary No clinic room for ANC services at the dispensary No quick service. Staff are too slow, patient have to wait for a long time No blood transfusion services for pregnant mothers and children Poor courtesy of nurses to patients including ANC mothers'	'No laboratory facilities at Kwamsisi dispensary' 'We do not have our own village dispensary. We either have to go to Kwamsisi or Magunga District Hospital' 'Sometimes we waste a lot of time when we go to Kwamsisi dispensary. You find so many people (i.e. pregnant women) at the hospital (meaning the dispensary) and the few staff you find there are tired and exhausted to give you service immediately for you to come back home earlier'	'Lack of blood transfusion services at Ngombezi dispensary' 'Lack of beds to admit pregnant mothers, especially if two or more coincidently attend at the dispensary at the same time' 'We are happy with the doctor, but...mhh.., as for the nurses...you may say why did I go there...'	'No village dispensary. We normally go to Ngombezi or Mandera, but the latter is too far for pregnant women' 'We need a health centre in this area. Even the Vice President was informed when he paid us a visit that we are facing an acute problem of not having a closer health centre here, while this village is one of the famous agricultural productive areas in this district' 'You can't imagine seeing a health worker shouting at you like a young person...'

### Attitudes about SP and IPTp among pregnant women

At times the pregnant women were uninformed or misinformed about the standard dosage of SP. A woman in Kwamsisi village stated: '*The drug we are given is sometimes little. Imagine, half a tablet for a child?*' In Tabora and Kitifu villages, the majority of the respondents alleged that one of the drawbacks linked to low acceptance of SP is a perceived relationship between SP side effects and HIV infection. Some women reported developing adverse reactions after using SP. Because of their belief and fear of the Steven-Johnson Syndrome, which was referred to as '*the burning of the skin*', it was openly asserted that some women threw away the SP tablets after leaving the dispensary. This problem was reported in all four study villages. One woman stated: "A*t least nowadays at Magunga *(i.e. the district hospital), *there is a new system of *DOT *intended to minimise the chances for those who could throw the drug away*". Similar statements were given by women in Tabora village. An example: "*Some women hesitate even to take the drugs at clinics*.*Some of them (and these are the majority) take them but either hide them and throw them later in the bush on their way home *or *when they reach home*". At Kwamsisi village other concerns were non-specific. One woman indicated: "*I do not like SP because it makes me feel bad*'. At Kitifu village it was argued that SP is an effective anti-malarial drug although some people find it less effective and opt for other drugs. When asked about choice of alternative drugs, Metakelfin^® ^was preferred. It was further argued that some women believed SP taken during pregnancy could cause abortion, whilst others decided to take smaller dosage than what is recommended. Other participants in the same FGD said that SP does not lower body temperature and that it causes one's (especially children's) body to weaken.

Some of the pregnant women said that SP prescribed at ANC clinics was meant for IPTp. They all seemed to share the general concept that SP was given because it was the recommended first line drug after the government had abandoned CQ. Of the 11 women discussants from Tabora village only four had prior information on the IPTp strategy. Those who seemed to be aware of it confirmed that they had received the information through attendance at ANC clinics and from announcements on the national radio. The research team also observed posters from the NMCP at the two dispensaries visited, which may have sensitized the community about the new treatment policy guidelines. At Kitifu village, mothers reported they had been given brochures with guidelines about the importance of using SP during pregnancy.

In the course of discussion at Tabora village, some of the women inquired whether the use of SP for IPTp was better than the use of insecticide-treated nets (ITNs) promoted by the government, NGOs and other agencies. All participants in the group felt that it would be better if the government constructed a health centre in their area due to the long travel distance to Ngombezi Dispensary located seven kilometres away.

### Suggestions on how to improve IPTp services

#### (a) Opinions from pregnant women

As illustrated in Table 2, community sensitization, health education activities and supervised uptake of SP by health staff were the key suggestions provided by pregnant women for improving IPTp services and increasing ANC attendance. The majority of the pregnant women felt that the community should be adequately informed about potential malaria-related risks and disadvantages resulting from failure to use the recommended antimalarial drugs, be it for IPTp or other purposes. In addition, people should be educated on potential dangers associated with self-medication without proper knowledge of the drugs taken or prior consultation with health personnel.

**Table 2:** Suggestions from pregnant women participating in FGDs about measures to improve the implementation of IPTp services. The suggestions represent short versions of explanations presented by FGD participants. "Yes" indicates that the suggestion was presented and agreed to be valid by several participants. "No" indicates that the suggestion was either not presented at all or was presented by one participant but did not receive any major support.

**Suggestions presented at FGDs**		**Kitifu**	**Kwakombo**	**Kwamsisi**	**Tabora**
1	Pregnant women should take SP under supervision by health staff to ensure that the drug is actually taken	Yes	No	No	No
2	Seminars to sensitize the community through acceptable local leaders	Yes	No	No	No
3	Radios and newspapers, though sometimes these are not reachable/affordable by most women	Yes	Yes	No	Yes
4	Posters at health facilities and in streets where many people pass by	No	No	No	Yes
5	Women lowly attend at local meetings, so few can benefit from such meetings	No	No	No	Yes
6	Health education and sensitization of ANC clients at health facilities	No	Yes	Yes	Yes
7	General public meetings	No	Yes	Yes	No
8	Women attended at ANC clinics to be given brochures with simple message about malaria treatment with SP	No	Yes	Yes	No

#### (b) Opinions from health care providers and CHMT members

Both the staff working at health facility level and CHMT members at district level admitted that since the IPTp strategy was a new malaria intervention recommended at national level, not all the health care service providers at peripheral health facility level were fully knowledgeable about it. For this reason, it was suggested that for the IPTp-services to be effectively delivered and actually utilized by target users, health care providers need to be trained well about IPTp service delivery. At the same time, more deliberate efforts should be made to sensitize pregnant women and the public at large about the strategy and importance of complying with the malaria treatment services as recommended. The issue of understaffing was highlighted as an impediment to delivering the desirable quality of service at most public health facilities, especially at dispensary level. It was added that unless the government addresses the problem of staff shortages in addition to providing other incentives such as timely staff promotion, remuneration and staff houses or housing allowances, there is a danger that staff compliance with the IPTp guidelines at most of the peripheral health facilities will be undermined.

## Discussion

The present study assessed the knowledge and attitudes of ANC service providers and users in relation to health care quality and malaria signs, symptoms, prevention and management with an emphasis on IPTp services and SP use. Although the level of knowledge about malaria was generally high among the participants, the risk factors associated with malaria in pregnancy were inadequately known by pregnant women. Several other observations also indicated that dissemination of information about malaria prevention and management to pregnant women attending ANC clinics was very poor. Two such observations are: 1) there was inadequate recognition that SP prescribed at the ANC facilities was for malaria preventive purposes and 2) there was uncertainty about whether the IPTp strategy was substituting or complementing the promotion of ITNs. Intensified health education and sensitization programmes at ANC facilities and community level appear to be useful measures for increasing acceptability and coverage of IPTp services among it users.

Substantial concern about the use of SP during pregnancy was expressed by pregnant women in the present study. Public concern about the use of SP for malaria treatment has also been observed in other recent unpublished studies carried out in Muheza District in Tanga Region, Tanzania (J. Massaga, personal communication) and in Mpwapwa District in Dodoma Region, Tanzania (S. Magesa, personal communication). Concern among users about perceived side effects of using SP during pregnancy has also been reported from a study in Kenya where 216 women of reproductive age in Kisumu District were interviewed [31]. This study observed that 96% of the respondents perceived malaria to be a problem during pregnancy and 74% believed that antimalarial drugs taken during pregnancy would be harmful to the pregnant woman and her unborn child. A study in Malawi showed that out of 809 pregnant women interviewed 37% believed that malaria would be harmful to both the mother and unborn child [31]. The massive concern expressed about the use of SP calls for intensified health education about the documented effects and side effects of SP and the possible consequences of avoiding this.

The observation that pregnant women occasionally throw away their SP tablets after leaving the ANC clinics justifies the need for measures to improve the implementation of the DOT approach for IPTp as was supported by the DMO and most of the study participants including pregnant women. To ensure equity to ANC such measures would include adequate and reliable supply of clean and safe water at the clinics rather than relying on requesting the pregnant women to bring their own water and cups.

The findings of the present study support the hypothesis that the success of any health intervention in terms of achieving its objectives cannot be merely justified by its efficacy but also depend on other factors such as the knowledge and skills of service providers and users, their motivation, attitudes, practices and a range of other socio-economic factors [32]. Pregnant women deciding to contact formal health facilities expect services of a certain desired quality besides receiving drug prescriptions. A country evaluation in Nepal indicated that basic improvement in the quality of health services delivered at health facilities was significantly more important to the target service users than the number of health posts *per se *[33]. The term "quality services" in this case included all services pertaining to customer care such as health workers' behaviour when dealing with their clients, waiting time at the service facility, availability of drugs and other basic services. Quality of health care also includes the technical competence/skills of a provider in delivering the care. Studies in India and elsewhere have found that good obstetric and antenatal care is fundamental to decreasing case fatality due to pregnancy related complications [34]. Although users' perceptions of the quality of care are highly subjective patients should rightly expect courtesy and attention from health service providers as well as proper clinical examination and medical advice [19,35]. Studies in Tanzania [1] and elsewhere [35] have observed a relationship between a user-perception of quality of care and their health care seeking behaviour for malaria and other illnesses and found that this has had negative implications on users' compliance with the recommended treatment procedures.

Staff motivation is an important factor to determine the quality of care, hence deserving attention by policy makers and other key decision-makers in the health system. Evidence shows that a well-functioning health system depends on the motivated work force, amongst other things [37]. In resource limited health system settings, however, solving problems related to staff de-motivation and poor quality of care will depend on cost and priority setting. Effective measures designed to improve staff motivation through systematic, long-term and well-planned in-service training and supportive supervision would contribute to the successful functioning of an intervention programme. This study and others indicate that the measures, if linked with proper performance monitoring of (and feedback to) service providers, including periodic user satisfaction assessments, are likely to affect the quality of care in a positive direction [37].

The desired quality of care cannot be fully realized when there is a shortage of health service personnel and supporting infrastructural facilities. In the present study the pregnant women at Kwamsisi complained about shortage of nurses and the lack of a rest room for pregnant women. Other complaints related to lack of blood transfusion services and laboratory and diagnostic facilities. Unless resolved, problems of this nature may directly or indirectly influence the health seeking behaviour of pregnant women who may ultimately decide to abandon the public health system and consult more private health care providers such as traditional health practitioners, retail drug sellers or private clinics. These may not be sufficiently competent or well monitored to ensure compliance with national guidelines.

Intensified health education and sensitization on malaria prevention and management were highlighted as major issues by pregnant women in this study. Health facilities and ANC clinics provide excellent bases for addressing the women about such matters. However, a woman's ability to act or seek for care may depend on several socio-cultural factors. The framework for health promotion should, therefore, be extended to the community and household levels mainly through extension health workers and volunteers involved with community-based health promotion, sensitization and mobilization. The mass media may also serve as a vehicle for dissemination of information although media accessibility and equity issues would have to be carefully considered in defining proper approaches. There is substantial literature documenting that poor people are reached less by public health interventions than affluent ones [27,36].

## Conclusion

This study identifies several potential constraints to the effective implementation of IPTp services in Tanzania. Based on that, it reveals that the effective delivery of antenatal and IPTp services may be influenced by the motivation of health service givers which by itself depends on the broad policy and health service environment in which they work. Timely and regular attendance to antenatal clinics by pregnant women and their positive perception of IPTp as a strategy and the drug recommended and on the quality of antenatal care are also important for the complete uptake of the recommended IPTp doses. Hence, the study gives suggestions for the strengthening of the IPTp strategy and increasing compliance and coverage. These relate to the need for intensifying health education and sensitization of beneficiaries and community members on the one hand and for addressing staff motivation factors and quality of care through training, supervision and monitoring of health service providers on the other. The latter recommendation is in line with the suggestion by other authors [38,39] that the scaling up of priority interventions requires significant investment in initial and continuous training. However, as the investment in all these aspects may be constrained by limited budget, it is important to think about all the possible fund raising options and lobbying towards making this a success. Besides aspects related to remuneration and other terms of employment, the working environment can also be considered in terms of the infrastructural conditions in which they work. Better health infrastructure, well equipped health facilities and hospitable staff are an attraction to service clients and policy makers should prioritize these issues in their strategic plans for antenatal care, IPTp and general health services. These conclusion and recommendations may be strengthened by a larger study to be conducted in Muheza district in 2005 that would be followed up by an evaluation of the IPTp strategy's implementation based on conclusions both in the baseline and main study. The conclusions and recommendations from the evaluation processes will add evidence to feedback stakeholders at district level and national-level policy makers on the way forward regarding the improved IPTp implementation approached in the country.

## Authors' contributions

GM participated in the study design, drafting and refining the data collection tools and actually participated in data collection, drafted the technical research report and the first and revised versions of the manuscript. PB provided technical input related to the study design and participated in the research report and manuscript writing. MK commented on the design of the data collection tools and participated in data collection. AK commented on the study design and approved the manuscript to be submitted for publication. In collaboration with PB, JI conceived the study, participated in the study design, data collection tools, data collection process and coordinated the fieldwork and manuscript writing.

## References

[B1] de Savigny D, Mayombana C, Mwageni E, Masanja H, Minhaj A, Mkilindi Y, Mbuya C, Kasale H, Reid G (2004). Care-seeking patterns for fatal malaria in Tanzania. Malar J.

[B2] Tami A, Mubyazi G, Talbert A, Mshinda H, Duchon S, Lengeler C (2004). Evaluation of Olyset insecticide treated nets distributed seven years previously in Tanzania. Malar J.

[B3] Olsen BE, Hinderaker SG, Bergsjo P, Lie RT, Olsen OHE, Gasheka P, Kvale G (2002). Causes and characteristics of maternal deaths in rural northern Tanzania. Acta Obstet Gynecol Scand.

[B4] Marchant T, Schellenberg-Armstrong JRM, Edgar T, Ronsmans C, Nathan R, Abdulla S, Mukasa O, Urasa H, Lengeler C (2002). Anaemia during pregnancy in southern Tanzania. Ann Trop Med Parasitol.

[B5] Kitua AY (1999). Antimalarial drug policy in Tanzania: making systemic change. Lancet.

[B6] Tarimo DS, Minjas JN, Bygbjerg CI (2001). Perception of chloroquine efficacy and alternative treatments for uncomplicated malaria in children in holoendemic area of Tanzania: implications for the change of treatment policy. Trop Med Int Health.

[B7] East African Network for Monitoring Antimalarial Treatment (EANMAT) (2001). Monitoring antimalarial drug resistance within national malaria control programmes: the EANMAT experience. Trop Med Int Health.

[B8] MoH Tanzania (2000). Antimalarial Drug Task Force, Tanzania. 2000. Implementation of a new Anti-malarial Treatment Policy in Tanzania: The rationale for change and guide to the process of policy implementation. Tanzania Health Research Bulletin.

[B9] Mutabingwa TK, Maxwell CA, Sia IG, Msuya FHM, Mkongewa S, Vannithone S, Curtis J, Curtis CF (2001). A trial of proguanil-dapsone in comparison with sulfadoxine-pyrimethamine for the clearance of Plasmodium falciparum infections in Tanzania. Trans R Soc Trop Med Hyg.

[B10] Mutabingwa T, Nzila A, Mberu E, Nduati E, Winstanley P, Hills E, Watkins W (2001). Chlorproguanil-dapsone for treatment of drug resistant falciparum malaria in Tanzania. Lancet.

[B11] UNICEF (2004). Malaria: a major cause of child death and poverty in Africa.

[B12] Malaria Consortium Tanzania Roll Back Malaria Consultative Mission Report,.

[B13] Schulman CE, Dorman E, Cutts F, Kawuondo K, Bulmer JN, Peshu N, Marsh K (1999). Intermittent sulfadoxine-pyrimethamine to prevent severe anaemia secondary to malaria in pregnancy: a randomised placebo controlled trial. Lancet.

[B14] Njagi J (2002). The effects of sulfadoxine-pyrimethamine intermittent treatment and pyrethroid impregnated bednets on malaria morbidity in pregnancy and birth-weight in Bondo district, Kenya. PhD Thesis.

[B15] Steketee RW, Wirima JJ, Campbell C (1996). The burden of malaria in pregnancy in malaria endemic areas. Am J Trop Med Hyg.

[B16] Verhoeff FH, Brabin BJ, Chimsuku L, Kazembe P, Russell WB, Broadhead RL (1998). An evaluation of the effects of intermittent SP treatment in Pregnancy on parasite clearance and risk of low birth-weight in rural Malawi. Ann Trop Med Parasitol.

[B17] Schultz L, Steketee RW, Parise M, Wirima JJ, Oloo A, Nahlen B A selection of Essays: Malaria during pregnancy: An antenatal intervention strategy whose time has come. IDRC Homepage.

[B18] Guyatt HL, Noor AM, Ochola SA, Snow RW (2004). Use of presumptive treatment and insecticide-treated bednets by pregnant women in four Kenyan districts. Trop Med Int Health.

[B19] Hanson K, Goodman C, Lines J, Meek S, Bradley D, Mills A (2004). The economics of malaria control interventions. Global Forum for Health Research.

[B20] Greenwood B (2000). Malaria-first, roll back expectations. Bull World Health Organ.

[B21] Attaran A Where did it go wrong?. Nature.

[B22] WHO/TDR Implementation research: bridging the gap between efficacy trials and application. TDR Newsletter No 71.

[B23] Bloland PB, Ettling M, Meek S (2000). Combination therapy for malaria in Africa: hype or hope?. Bull World Health Organ.

[B24] Garner P, Gulmezoglu AA (2003). Prevention versus treatment for malaria in pregnant women. Cochrane Database Systems Review.

[B25] Greenwood B (2004). The use of antimalarial drugs to prevent malaria in the populations of malaria endemic areas. Am J Trop Med Hyg.

[B26] Breman JG, Alilio MS, Mills A (2004). Conquering the intolerable burden of malaria: What is needed: A summary. Am J Trop Med Hyg.

[B27] Barat LM, Palmer N, Basu S, Worrall E, Hanson K, Mills A (2004). Do malaria control interventions reach the poor? A view through the equity lens. Am J Trop Med Hyg.

[B28] WHO & UNICEF The Africa Malaria Report on Malaria during pregnancy. http://http:www.rbm.who.int/amd2003/amr2003/ch4.htm/pdf.

[B29] van Eijk AM, Ayisi AG, tel Kuile FO, Otieno JA, Misore Odondi JO, Rose DH, Kager PA, Steketee RW, Nahlen BL (2004). Implementation of intermittent preventive treatment with sulfadoxine-pyrimethamine for control of malaria in pregnancy in Kisumu, western Kenya,. Trop Med Int Health.

[B30] Hotz TH, Kachur SP, Roberts JM, Marum LH, Mkandala C, Macheso A (2004). Use of antenatal services and IPT for malaria among pregnant women in Blantyre, Malawi District. Trop Med Int Health.

[B31] Ndyomugenyi R, Neema S, Magnussen P (1998). The use of informal services for antenatal care and malaria treatment in rural Uganda. Health Policy & Planning.

[B32] Acharya LB, Cleland J (2000). Maternal and child health services in rural Nepal. Health Policy & Planning.

[B33] Ramarao S, Caleb L, Khan ME, Townsend JW (2001). Safer maternal health in rural Uttar Praesh: do primary health services contribute?. Health Policy & Planning.

[B34] Hanson K Good economics – implementing cost-effective strategies against malaria. DFID Homepage, article dated 24th January 2004 under the heading 'Communicating health development research'.

[B35] Goodman C, Coleman P, Mills A (2003). Economic analysis of malaria control in sub-Saharan Africa. Global Forum for Health Research.

[B36] Dieleman M, Cuong PV, Anh LV, Martineau T (2003). Identifying factors for job motivation of rural health workers in North Viet Nam. Human Resources for Health.

[B37] Wyss K (2004). An approach to clarifying human resources constraints to attaining health-related Millennium Development Goals. Human Resources for Health.

[B38] WHO, World Bank (2003). High-level Forum on the Health Millenium Development Goals: Improving Workforce Performance: Issues for Discussion: Session 4.

[B39] Rønn A, Msangeni H, Mhina J (1996). High level of resistance of *P. falciparum *to sulfadoxine-pyrimethamine in children in Tanzania. Trans R Soc Hyg Trop Med.

[B40] Ministry of Health (2004). Focused antenatal care malaria and syphilis during pregnancy: orientation package for service providers.

